# Assessment of Prevalence and Characteristics of Valvular Heart Disease: A Cross‐Sectional Study From Yazd Health Study

**DOI:** 10.1002/hsr2.72306

**Published:** 2026-04-06

**Authors:** Hasan Haghani Nejad, Sedighe Sadeghi, Mohammad Hossein Soltani, Elahe Zare, Forough Sadat Tabatabaei, Mojtaba Jokar

**Affiliations:** ^1^ Yazd Cardiovascular Research Center, Non‐Communicable Diseases Research Institute Shahid Sadoughi University of Medical Sciences Yazd Iran

**Keywords:** echocardiography, reduced ejection fraction, hypertension, mitral valve prolapse, valvular heart disease

## Abstract

**Background and Aims:**

Valvular heart diseases (VHDs) significantly contribute to global cardiovascular mortality and morbidity, with a shift in epidemiology from rheumatic heart disease (RHD) in developing nations to degenerative valvular diseases in aging populations of industrialized countries. We aim to evaluate the prevalence and pathological patterns of VHDs in individuals over 40 in Yazd, Iran, using high‐quality echocardiographic evaluations to inform public health strategies and optimize resource allocation for VHD prevention and management.

**Methods:**

This cross‐sectional study involved 600 randomly selected individuals aged 40 and older from the Yazd Health Study (YaHS), conducted at Afshar Hospital in Yazd, Iran, from September 2023 to May 2024. Participants underwent echocardiography to assess valvular health, with demographic and comorbidity data collected from medical records. Echocardiographic evaluations were performed using a VIVID 4 ultrasound device, following guidelines from the American Society of Echocardiography.

**Results:**

Among the 600 participants, 35 (5.8%) were diagnosed with VHD, with mitral valve prolapse (MVP) being the most common (28.6%), followed by tricuspid regurgitation (TR) (20%), mitral regurgitation (MR) (11.4%), aortic regurgitation (AR) (11.4%), and MS (8.6%). Hypertension (HTN) was significantly more prevalent in VHD patients (71.4%) compared to those without VHD (35.2%), and reduced ejection fraction (EF < 45%) was also more common in the VHD group. Degenerative changes were the leading cause of VHD, making up 57.1% of cases, while HTN and reduced EF were identified as significant independent risk factors for VHD.

**Conclusion:**

This study reveals a significant prevalence of VHD in the YaHS, with HTN and reduced EF identified as key risk factors. MVP was the most common type of VHD, followed by TR, MR, and AR. The findings emphasize the need for early management of HTN and heart failure (HF) to reduce the impact of VHD.

## 1 Introduction

Valvular heart diseases (VHDs) are a significant contribution to worldwide cardiovascular mortality and morbidity, as well as imposing enormous healthcare burdens [1, 2]. These conditions include various pathological anomalies of the heart valves, such as regurgitation, stenosis, or a combination of both, which affect hemodynamic function [2]. The epidemiology of VHDs has undergone a notable transformation over the past several decades, with rheumatic heart disease (RHD) declining in prevalence in industrialized nations while degenerative valvular diseases have emerged as dominant etiologies, particularly among aging populations [3, 4, 5]. In contrast, RHD remains a significant public health concern in developing nations, disproportionately affecting younger individuals during their most productive years [6, 7].

Echocardiography remains the gold standard in diagnosing and following VHDs, offering critical insights into anatomical and functional impairments [8]. Nonetheless, the worldwide incidence of VHD is probably underestimated, especially in low‐resource environments, due to limited access to advanced diagnostic instruments [9, 10, 11]. Recent studies have emphasized inequalities in the occurrence and consequences of VHD among genders, regions, and socioeconomic groups [10].

Data on the prevalence of VHDs in developing countries, including Iran, need to be more extensive. Existing studies have primarily focused on ischemic heart disease (IHD) or heart failure (HF), neglecting the burden of non‐rheumatic valvular heart diseases (NRVDs) [7, 12, 13]. Furthermore, shifts in etiology driven by increasing life expectancy and improved healthcare access have likely altered the patterns of VHDs, emphasizing the need for region‐specific epidemiological data [13, 14].

This study aimed to evaluate the prevalence and pathological patterns of VHDs in individuals over 40 years old in a random population of Yazd, Iran. By utilizing high‐quality echocardiographic evaluations, we seek to provide robust evidence to inform public health strategies and optimize resource allocation to prevent and manage VHDs.

## 2 Methods

### 2.1 Study Population

This cross‐sectional study included 600 individuals, randomly selected, aged 40 years and more, registered in the Yazd Health Study (YaHS) and was conducted from September 2023 to May 2024 at Afshar Hospital, a university‐affiliated tertiary medical center in Yazd, Iran. The participants were invited to this medical center to perform echocardiography. The YaHS participants were randomly included in the study. YaHS was a study aimed at determining the prevalence of non‐communicable diseases and associated risk factors in the Greater Yazd Area. The methodology details were published elsewhere. In an overview, the YaHS population frame consisted of Yazd Greater Area adults aged 20–69. Ten thousand participants (200 clusters of 50) were selected using a two‐step cluster sampling procedure [15]. Demographic information, including age, gender, and history of comorbidities such as diabetes mellitus (DM), hypertension (HTN), and IHD, was obtained from the patients' medical records.

### 2.2 Ethical Considerations

The study was approved by the ethical committee of Shahid Sadoughi University of Medical Sciences (IR.SSU.MEDICINE.REC.1401.126). All study procedures were conducted according to the Declaration of Helsinki, and informed consent was obtained from all patients prior to the study.

### 2.3 Echocardiography Evaluation

All patients underwent Transthoracic echocardiography using a VIVID 4 ultrasound device (GE Medical Systems) at Afshar Hospital, Yazd, Iran [16]. The assessment of valvular stenosis and regurgitation was carried out as recommended according to the guidelines of the American Society of Echocardiography [17]. Valve regurgitation was assessed based on the quantitative parameters, including regurgitant volume and effective regurgitant orifice area, and supported by semiquantitative methods like vena contracta width and pressure half time. Valve stenosis severity was assessed based on the peak gradient and velocity, mean gradient and velocity, and valve area. All studies were performed and reported by an experienced sonographer.

### 2.4 Statistical Analysis

The SPSS version 23 for Windows (SPSS Inc., Chicago, IL, United States) software package was used to analyze all the data. Qualitative variables were presented with frequency and percentage, and compared between groups by *χ* ^2^ test. An independent *t*‐test was used to compare quantitative variables expressed with mean ± standard deviation. The univariate effect of factors on VHD was evaluated by univariate regression analysis. All possible confounders with a univariate *p* value< 0.1 were used in the initial iteration of a multivariate regression model. The variables included in the multivariable model were selected based on clinical findings and prior literature. The findings were presented in terms of odds ratios (OR), along with corresponding 95% confidence intervals (95% CI). A two‐sided *p* value of less than 0.05 was considered statistically significant.

## 3 Results

**Figure 1:**
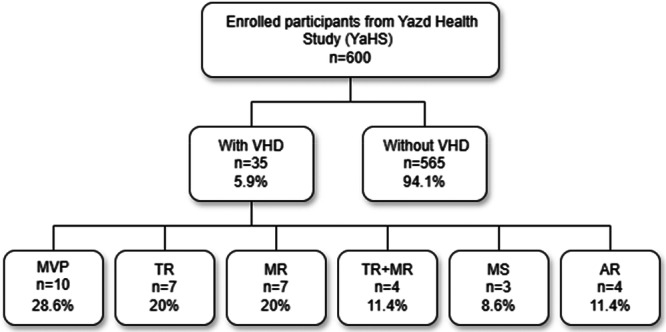
Distribution of the different types of valvular heart disease. Abbreviations: AR, aortic regurgitation; MR, mitral regurgitation; MS, mitral stenosis; MVP, mitral valve prolapse; TR, tricuspid regurgitation.

Among the 600 participants, 35 (5.8%) were diagnosed with VHD. The most common type was mitral valve prolapse (MVP), observed in 28.6% of cases. This was followed by tricuspid regurgitation (TR), seen in 20% of cases; mitral regurgitation (MR), present in 11.4%; aortic regurgitation (AR), observed in 11.4%; and mitral stenosis (MS), found in 8.6% of cases (Figure 1).

The baseline characteristics of participants with and without VHD are summarized. HTN was significantly more prevalent among VHD patients than without VHD (71.4% vs. 35.2%, *p* < 0.001). Additionally, reduced ejection fraction (EF < 45%) was more frequent in the VHD group (11.4% vs. 2.5%, *p* < 0.001). There were no significant differences in age, sex, DM, or IHD between the two groups (Table 1).

**Table 1:** Characters of the study population with and without valvular heart disease.

Variable	Total (*N* = 600)	VHD (*N* = 35)	No VHD (*N* = 565)	*p* value
Age (years)	58.84 ± 10.63	59.83 ± 10.68	58.78 ± 10.68	0.57*
40–49	149 (24.8)	8 (22.9)	141 (25.0)	0.6**
50–59	152 (25.3)	6 (17.1)	146 (25.8)	
60–69	194 (32.3)	14 (40.0)	180 (31.9)	
≥ 70	105 (17.5)	7 (20.0)	98 (17.3)	
Sex, *n* (%)				0.8**
Male	287 (47.8)	16 (45.7)	271 (48.0)	
Female	313 (52.2)	19 (54.3)	294 (52.0)	
DM, *n* (%)	176 (29.3)	12 (34.3)	164 (29.0)	0.5**
IHD, *n* (%)	58 (9.7)	5 (14.3)	53 (9.4)	0.34**
HTN, *n* (%)	224 (37.3)	25 (71.4)	199 (35.2)	< 0.001**
EF category, *n* (%)				< 0.001**
≥ 55	567 (94.5)	29 (82.9)	538 (95.2)	
54–45	15 (2.5)	2 (5.7)	13 (2.3)	
< 45	18 (3.0)	4 (11.4)	14 (2.5)	

*Note:* Data are expressed as mean ± SD or *n* (%).

Abbreviations: DM, diabetes mellitus; EF, ejection fraction; HTN, hypertension; IHD, ischemic heart disease.

* Independent *t*‐test,

** *χ* ^2^, or Fisher's exact test, was used.

Degenerative changes were the leading cause of VHD, accounting for 57.1% of cases, followed by inflammatory causes (14.3%), rheumatic disease (11.4%), endocarditis (11.4%), and ischemic changes (5.7%) (Table 2).

**Table 2:** Prevalence of different etiologies of valvular heart diseases.

	Frequency	Percent
Degenerative	20	57.1
Rheumatic	4	11.4
Endocarditis	4	11.4
Inflammatory	5	14.3
Ischemic	2	5.7

**Figure 2:**
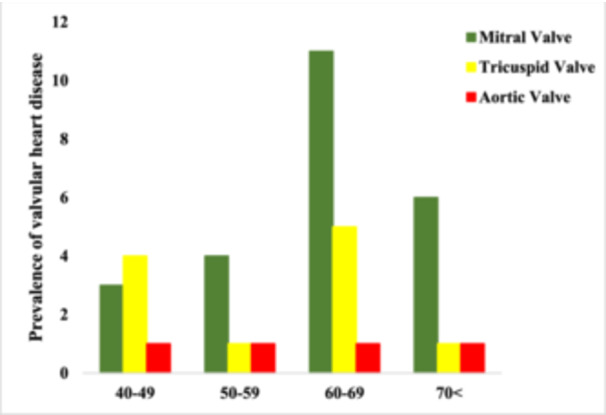
Prevalence of mitral, tricuspid, and aortic valve diseases according to age.

The prevalence of mitral, tricuspid, and aortic valve involvement increased with age, particularly in degenerative and inflammatory etiologies (Figure 2).

HTN and reduced EF were identified as significant independent risk factors for VHD. HTN was associated with an increased risk of VHD in both univariate analysis (OR: 4.59, 95% CI: 2.16–9.76, *p* = 0.001) and multivariate analysis (OR: 4.22, 95% CI: 1.96–9.08, *p* = 0.001). Similarly, EF < 45% significantly increased the risk of VHD in univariate (OR: 5.3, 95% CI: 1.64–17.11, *p* = 0.005) and multivariate analyses (OR: 4, 95% CI: 1.19–13.44, *p* = 0.025). No significant associations were observed for age, sex, DM, or IHD (Table 3).

**Table 3:** Univariate and multivariate analysis: risk factors for valvular heart disease.

	Univariate			Multivariate		
	OR	95% CI	*p* value	OR	95% CI	*p* value
Age	1.009	0.97–1.04	0.57			
Sex, female (ref = male)	1.09	0.55–2.17	0.8			
DM	1.27	0.62–2.62	0.5			
IHD	1.6	0.59–4.32	0.35			
HTN	4.59	2.16–9.76	0.001	4.22	1.96–9.08	0.001
EF category (Ref ≥ 55)						
54–45	2.84	0.61–13.25	0.18	1.69	0.35–8.08	0.5
< 45	5.3	1.64–17.11	0.005	4	1.19–13.44	0.025

*Note:* The results are expressed as odds ratio and 95% confidence intervals.

## 4 Discussion

VHD is a prevalent cardiovascular disorder worldwide, influenced by a range of risk factors, including genetic predisposition, advancing age, male gender, HTN, elevated cholesterol levels, tobacco use, diabetes, adrenal insufficiency, rheumatic conditions, and infectious endocarditis [18]. Although VHD is less common than coronary artery disease (CAD) and HF in developed countries, the establishment of comprehensive guidelines remains essential due to the frequent necessity for invasive procedures such as valve repair or replacement (VR) [19]. Our study, conducted in an Iranian cardiac center, aimed to explore the prevalence and characteristics of VHD, an area that remains understudied in the context of the Iranian population. We found a 5.8% prevalence of VHD among 600 participants from Yazd, which aligns with findings from similar studies globally, though some reports show higher rates, particularly in aging populations [20]. We contrast our findings with previous evidence to enhance understanding. The most common form of VHD in our cohort was MVP, found in 28.6% of cases. This finding mirrors earlier studies that suggest MVP is prevalent in approximately 2‐3% of the general population, yet our study reveals it to be more common in the Yazd population [21, 22]. This could reflect regional variations or differences in the study sample's age and health profile.

MVP is characterized by the displacement of mitral leaflets into the left atrium by more than 2 mm, with its primary causes being diffuse myxomatous degeneration (Barlow disease) and fibroelastic degeneration linked to aging [22]. In our findings, other valvular abnormalities, including TR and MR, underscore the diverse presentation of VHD in the general populace. Significant TR is particularly concerning, as it is associated with debilitating symptoms and increased mortality risk [23]. An analysis of 714,368 patient records in the United States revealed that VHD is most prevalent among women, with TR occurring at a rate of 7.1%, followed by MR at 6.5%, aortic stenosis (AS) at 4.1%, AR at 2.3%, and mitral stenosis at 0.5% [24]. A notable observation from our study was the significant association between HTN and VHD, with HTN patients showing a higher prevalence of VHD compared to those without HTN. This aligns with previous studies, such as Saadeh et al., which highlight the detrimental role of HTN in cardiovascular remodeling, particularly in relation to valvular pathology [25]. In our cohort, HTN emerged as a significant independent risk factor for VHD in both univariate and multivariate analyses. This supports the clinical understanding that HTN‐induced structural changes predispose individuals to valve dysfunction. Our results emphasize the need for early identification and management of HTN to mitigate the risk of VHD [26, 27]. The relationship between reduced ejection fraction (EF) and VHD is another critical finding of our study. We observed that reduced EF was significantly more prevalent among patients with VHD, which resonates with research by Najafi et al., who found that ventricular dysfunction can precede or result from valvular abnormalities, influencing long‐term patient outcomes [28]. This connection underscores the importance of evaluating both ventricular function and valve status in patients with cardiovascular diseases, especially those at risk for HF. Similarly, Wu et al. highlighted VHD as a prognostic factor in HF patients, suggesting that valvular abnormalities can influence the progression and outcomes of ventricular dysfunction [29]. In terms of etiological factors, our study primarily identified degenerative changes as the leading cause of VHD, a finding consistent with the aging population and the progressive nature of valvular degeneration [30, 31]. This emphasizes the increasing burden of degenerative VHD as the global population ages. Moreover, inflammation, rheumatic disease, endocarditis, and ischemic changes were also identified as contributing factors in our cohort, aligning with findings from other studies that recognize the multifactorial origins of VHD. Cai et al. linked immune‐mediated inflammatory diseases (IMIDs) to an increased risk of VHD, exacerbated by both traditional cardiovascular risk factors and chronic inflammation [32]. Chronic inflammation can lead to valve fibrosis, driven by mediators like transforming growth factor‐β [33]. However, it is essential to note that degenerative causes may become more pronounced with age, particularly in high‐income countries, suggesting the need for targeted prevention strategies in these populations [34]. Aaramaa et al. emphasized that cardiovascular diseases are the most common comorbidity in rheumatic conditions, significantly elevating mortality risk beyond traditional cardiovascular factors [35]. The WHO reported 33.4 million cases of rheumatic valvular heart disease, with annual mortality expected to rise due to autoimmune and inflammatory responses to rheumatic fever [36]. Infective endocarditis, marked by substantial morbidity and mortality, disproportionately affects men and high‐risk groups, including those with previous infections or prosthetic heart valves [37]. The role of proteoglycans in modulating inflammation and cardiac remodelling underscores the intricate relationship between age, inflammation, and VHD [38]. Our analysis confirmed univariate and multivariate models, identifying HTN and reduced EF as significant independent risk factors for VHD. The odds ratios obtained provide compelling evidence for these clinical parameters in the pathogenesis of VHD. Conversely, the lack of significant associations with age, sex, diabetes mellitus, or ischemic heart disease suggests that these factors do not independently predict VHD in our cohort. While the prevalence of diabetes in VHD varies by etiology, peaking at 28.7% in ischemic VHD due to mechanisms like oxidative stress and endothelial dysfunction, which contribute to CAD and its subsequent complications [39].

Recent evidence has underscored an important morphological relationship between MVP and thoracic skeletal configuration, particularly a concave‐shaped anterior chest wall and varying degrees of pectus excavatum or other anterior chest wall deformities. These structural variations may alter cardiac geometry and mitral annular dynamics, contributing to leaflet displacement and valvular dysfunction. Therefore, integrating anthropometric or chest wall assessments in echocardiographic screening could improve the precision of MVP diagnosis and risk stratification [40]. However, it is important to note that this study did not explore the potential impact of these chest wall deformities on MVP presentation. Future clinical evaluations should incorporate such morphologic considerations to better identify individuals predisposed to MVP and to refine preventive and interventional strategies.

## 5 Limitations

This study has several limitations. The present study is cross‐sectional and cannot be used to determine cause‐and‐effect relationships. In addition, the effect of other potential confounders, such as lifestyle factors or genetic predispositions, on VHD was not examined in this study, which may lead to worse outcomes in patients with VHD. Finally, the long‐term outcomes of VHD were not examined in this study, and future cohort studies are needed to investigate the outcomes of VHD further.

## 6 Conclusion

In conclusion, this study highlights the prevalence and risk factors associated with VHD in a sample population from the YaHS. HTN and reduced EF were identified as significant risk factors for VHD. MVP as the most common type of VHD, followed by other conditions such as TR, MR, and AR. These findings emphasize the need for early detection and management of HTN and HF to reduce the burden of VHD. However, further studies with larger populations are needed to confirm these findings, explore the long‐term consequences of VHD, and explore the impact of these risk factors.

## Author Contributions

**Hasan Haghani Nejad:** conceptualization, methodology, data curation, project administration, writing – review and editing. **Sedighe Sadeghi:** writing – review and editing, writing – original draft, and data curation. **Mohammad Hossein Soltani:** data curation, validation, and investigation. **Elahe Zare:** validation, investigation. **Forough Sadat Tabatabaei:** software, data curation, investigation, formal analysis, visualization, writing – original draft, writing – review and editing. **Mojtaba Jokar:** writing – review and editing, supervision, and investigation.

## Funding

The authors have nothing to report.

## Ethics Statement

The study was approved by the Medical Ethics Committee of Ahvaz Jundishapur University of Medical Sciences (IR.AJUMS.REC.1402.609) and registered in the Iranian Clinical Trials Registry (IRCT20240213060989N1). The study was conducted in accordance with the principles of the Declaration of Helsinki for medical research.

## Consent

Informed consent was obtained from eligible individuals. The data collection process ensured complete anonymity, with no personally identifying information included in the individual data.

## Conflicts of Interest

The authors declare no conflicts of interest.

## Transparency Statement

The lead author Forough Sadat Tabatabaei affirms that this manuscript is an honest, accurate, and transparent account of the study being reported; that no important aspects of the study have been omitted; and that any discrepancies from the study as planned (and, if relevant, registered) have been explained.

## Data Availability

The data sets used and/or analysed during the current study are available from the corresponding author on reasonable request.

## References

[1] C. Manjunath , P. Srinivas , K. Ravindranath , and C. Dhanalakshmi , “Incidence and Patterns of Valvular Heart Disease in a Tertiary Care High‐Volume Cardiac Center: A Single Center Experience,” Indian Heart Journal 66, no. 3 (2014): 320–326. 24973838 10.1016/j.ihj.2014.03.010 PMC4121759

[2] J. S. Aluru , A. Barsouk , K. Saginala , P. Rawla , and A. Barsouk , “Valvular Heart Disease Epidemiology,” Medical Sciences 10, no. 2 (2022): 32. 35736352 10.3390/medsci10020032 PMC9228968

[3] S. S. Virani , A. Alonso , H. J. Aparicio , et al., “Heart Disease and Stroke Statistics–2021 Update: A Report From the American Heart Association.” 2021. 10.1161/CIR.0000000000000950 PMC13036842 33501848

[4] B. Iung and A. Vahanian , “Epidemiology of Acquired Valvular Heart Disease,” Canadian Journal of Cardiology 30, no. 9 (2014): 962–970. 24986049 10.1016/j.cjca.2014.03.022

[5] B. Prendergast , P. MacCarthy , and S. Ray , “Changing Epidemiology and Natural History of Valvular Heart Disease,” Clinical Medicine 10, no. 2 (2010): 168–171. 20437994 10.7861/clinmedicine.10-2-168 PMC4952095

[6] J. R. Carapetis , “Rheumatic Heart Disease in Developing Countries,” New England Journal of Medicine 357, no. 5 (2007): 439–441. 17671252 10.1056/NEJMp078039

[7] D. A. Watkins , C. O. Johnson , S. M. Colquhoun , et al., “Global, Regional, and National Burden of Rheumatic Heart Disease, 1990–2015,” New England Journal of Medicine 377, no. 8 (2017): 713–722. 28834488 10.1056/NEJMoa1603693

[8] J. Marangou , A. Beaton , T. O. Aliku , M. C. P. Nunes , N. Kangaharan , and B. Reményi , “Echocardiography in Indigenous Populations and Resource Poor Settings,” Heart, Lung and Circulation 28, no. 9 (2019): 1427–1435. 10.1016/j.hlc.2019.05.176 31272827

[9] S. Coffey , A. R. Harper , B. J. Cairns , I. S. Roberts , and B. D. Prendergast , “Clinical Information has Low Sensitivity for Postmortem Diagnosis of Heart Valve Disease,” Heart 103, no. 13 (2017): 1031–1035. 28183793 10.1136/heartjnl-2016-310718

[10] J. T. DesJardin , J. Chikwe , R. T. Hahn , J. W. Hung , and F. N. Delling , “Sex Differences and Similarities in Valvular Heart Disease,” Circulation Research 130, no. 4 (2022): 455–473. 35175844 10.1161/CIRCRESAHA.121.319914 PMC8869851

[11] S. Coffey , R. Roberts‐Thomson , A. Brown , et al., “Global Epidemiology of Valvular Heart Disease,” Nature Reviews Cardiology 18, no. 12 (2021): 853–864. 34172950 10.1038/s41569-021-00570-z

[12] J. Arabloo , N. Omidi , A. Rezapour , A. Sarabi Asiabar , S. Mojtaba Ghorashi , and S. Azari , “The Burden of Nonrheumatic Valvular Heart Diseases in Iran Between 1990 and 2017: Results From the Global Burden of Disease Study 2017,” International Journal of Cardiology. Heart & Vasculature 39 (2022): 100956. 35402692 10.1016/j.ijcha.2022.100956 PMC8984628

[13] J. Rwebembera , W. Manyilirah , Z. W. Zhu , et al., “Prevalence and Characteristics of Primary Left‐Sided Valve Disease in a Cohort of 15,000 Patients Undergoing Echocardiography Studies in a Tertiary Hospital in Uganda,” BMC Cardiovascular Disorders 18 (2018): 82. 29728065 10.1186/s12872-018-0813-5 PMC5935941

[14] H. Boudoulas , M. Vavuranakis , and C. F. Wooley , “Valvular Heart Disease: The Influence of Changing Etiology on Nosology,” Journal of Heart Valve Disease 3, no. 5 (1994): 516–526. 8000586

[15] M. Mirzaei , A. Salehi‐Abargouei , M. Mirzaei , and M. A. Mohsenpour , “Cohort Profile: The Yazd Health Study (YaHS): A Population‐Based Study of Adults Aged 20–70 Years (Study Design and Baseline Population Data),” International Journal of Epidemiology 47, no. 3 (2018): 697–698h. 29186588 10.1093/ije/dyx231

[16] S. Sadeghi , M. Jokar , S. M. S. H. Tezerjani , et al., “Electrocardiography Changes and Different Stages of Heart Failure in Central Iran: A Cross‐Sectional Study From Yazd Health Study,” Health Science Reports 7, no. 4 (2024): e2011. 38590915 10.1002/hsr2.2011 PMC11000134

[17] H. Baumgartner , J. Hung , J. Bermejo , et al., “Recommendations on the Echocardiographic Assessment of Aortic Valve Stenosis: A Focused Update From the European Association of Cardiovascular Imaging and the American Society of Echocardiography,” European Heart Journal—Cardiovascular Imaging 18, no. 3 (2017): 254–275. 28363204 10.1093/ehjci/jew335

[18] B. Taghizadeh , L. Ghavami , H. Derakhshankhah , et al., “Biomaterials in Valvular Heart Diseases,” Frontiers in Bioengineering and Biotechnology 8 (2020): 529244. 33425862 10.3389/fbioe.2020.529244 PMC7793990

[19] S. Azari , A. Rezapour , N. Omidi , et al., “A Systematic Review of the Cost‐Effectiveness of Heart Valve Replacement With a Mechanical Versus Biological Prosthesis in Patients With Heart Valvular Disease,” Heart Failure Reviews 25, no. 3 (2020): 495–503. 31823104 10.1007/s10741-019-09897-9

[20] A. Marciniak , K. Glover , and R. Sharma , “Cohort Profile: Prevalence of Valvular Heart Disease in Community Patients With Suspected Heart Failure in UK,” BMJ Open 7, no. 1 (2017): e012240. 10.1136/bmjopen-2016-012240 PMC5278264 28131996

[21] C. Delwarde , R. Capoulade , J. Mérot , et al., “Genetics and Pathophysiology of Mitral Valve Prolapse,” Frontiers in Cardiovascular Medicine 10 (2023): 1077788. 36873395 10.3389/fcvm.2023.1077788 PMC9978496

[22] A. Esposito , M. Gatti , M. G. Trivieri , et al., “Imaging for the Assessment of the Arrhythmogenic Potential of Mitral Valve Prolapse,” European Radiology 34, no. 7 (2024): 4243–4260. 38078997 10.1007/s00330-023-10413-9 PMC11164824

[23] R. T. Hahn , R. Makkar , V. H. Thourani , et al., “Transcatheter Valve Replacement in Severe Tricuspid Regurgitation,” New England Journal of Medicine 392, no. 2 (2025): 115–126. 39475399 10.1056/NEJMoa2401918

[24] R. T. Hahn , J. Lindenfeld , M. Böhm , et al., “Tricuspid Regurgitation in Patients With Heart Failure and Preserved Ejection Fraction,” Journal of the American College of Cardiology 84, no. 2 (2024): 195–212. 38960514 10.1016/j.jacc.2024.04.047

[25] R. Saadeh , B. Abu Jaber , T. Alzuqaili , S. Ghura , T. Al‐Ajlouny , and A. M. Saadeh , “The Relationship of Atrial Fibrillation With Left Atrial Size in Patients With Essential Hypertension,” Scientific Reports 14, no. 1 (2024): 1250. 38218895 10.1038/s41598-024-51875-1 PMC10787833

[26] X.‐Z. Hou , Y.‐F. Lv , Y.‐S. Li , et al., “Association Between Different Insulin Resistance Surrogates and All‐Cause Mortality in Patients With Coronary Heart Disease and Hypertension: NHANES Longitudinal Cohort Study,” Cardiovascular Diabetology 23, no. 1 (2024): 86. 38419039 10.1186/s12933-024-02173-7 PMC10903030

[27] M. Lu , D. Li , Y. Hu , et al., “Persistence of Severe Global Inequalities in the Burden of Hypertension Heart Disease From 1990 to 2019: Findings From the Global Burden of Disease Study 2019,” BMC Public Health 24, no. 1 (2024): 110. 38184560 10.1186/s12889-023-17573-9 PMC10771693

[28] M. S. Najafi , S. Nematollahi , A. Vakili‐Basir , et al., “Predicting Outcomes in Patients With Low Ejection Fraction Undergoing Coronary Artery Bypass Graft,” International Journal of Cardiology. Heart & Vasculature 52 (2024): 101412. 38694271 10.1016/j.ijcha.2024.101412 PMC11060952

[29] N. Wu , X. Lang , Y. Zhang , B. Zhao , and Y. Zhang , “Predictors and Prognostic Factors of Heart Failure With Improved Ejection Fraction,” Reviews in Cardiovascular Medicine 25, no. 8 (2024): 280. 39228475 10.31083/j.rcm2508280 PMC11367010

[30] X. Guo , Z. Li , T. Long , et al., “Physical Frailty and the Risk of Degenerative Valvular Heart Disease,” Innovation in Aging 8, no. 8 (2024): igae062. 39131201 10.1093/geroni/igae062 PMC11310592

[31] Y. Fei , J. J. Jo , S. Chen , et al., “Quantifying Cardiac Dysfunction and Valvular Heart Disease Associated With Subretinal Drusenoid Deposits in Age‐Related Macular Degeneration,” European Journal of Ophthalmology 34, no. 6 (2024): 2038–2044. 38545630 10.1177/11206721241244413

[32] D. Cai , Z. Zheng , J. Hu , Y. Fu , Y. Song , and J. Lian , “Immune‐Mediated Inflammatory Diseases and the Risk of Valvular Heart Disease: A Mendelian Randomization Study,” Clinical Rheumatology 43, no. 1 (2024): 533–541. 37505304 10.1007/s10067-023-06693-7

[33] A. M. Small , K. E. Yutzey , B. A. Binstadt , et al., “Unraveling the Mechanisms of Valvular Heart Disease to Identify Medical Therapy Targets: A Scientific Statement From the American Heart Association,” Circulation 150, no. 6 (2024): e109–e128. 38881493 10.1161/CIR.0000000000001254 PMC11542557

[34] G. Karthikeyan , M. Ntsekhe , S. Islam , et al., “Mortality and Morbidity in Adults With Rheumatic Heart Disease,” Journal of the American Medical Association 332, no. 2 (2024): 133–140. 38837131 10.1001/jama.2024.8258 PMC11154374

[35] H.‐K. Aaramaa , N. Mars , M. Helminen , et al., “Risk of Cardiovascular Comorbidities before and After the Onset of Rheumatic Diseases,” Seminars in Arthritis and Rheumatism 65 (2024): 152382. 38308930 10.1016/j.semarthrit.2024.152382

[36] S. Shafi , S. Aouabdi , Z. A. Taher , et al., “The Prevalence and Predictors of Atherosclerotic Coronary Artery Disease in Rheumatic and Non‐Rheumatic Valvular Heart Disease Patients,” Cureus 16, no. 3 (2024): e57317. 38690477 10.7759/cureus.57317 PMC11060012

[37] E. Havers‐Borgersen , L. Østergaard , C. K. Holgersson , et al., “Infective Endocarditis With or Without Congenital Heart Disease: Clinical Features and Outcomes,” European Heart Journal 45, no. 44 (2024): 4704–4715. 39217474 10.1093/eurheartj/ehae548

[38] S. Linna‐Kuosmanen , E. Schmauch , K. Galani , et al., “Transcriptomic and Spatial Dissection of Human Ex Vivo Right Atrial Tissue Reveals Proinflammatory Microvascular Changes in Ischemic Heart Disease,” Cell Reports Medicine 5, no. 5 (2024): 101556. 38776872 10.1016/j.xcrm.2024.101556 PMC11148807

[39] Q. Lu , J. Lv , Y. Ye , et al., “Prevalence and Impact of Diabetes in Patients With Valvular Heart Disease,” iScience 27, no. 3 (2024): 109084. 38375234 10.1016/j.isci.2024.109084 PMC10875155

[40] A. Sonaglioni , G. L. Nicolosi , and M. Lombardo , “The Relationship Between Mitral Valve Prolapse and Thoracic Skeletal Abnormalities in Clinical Practice: A Systematic Review,” Journal of Cardiovascular Medicine 25, no. 5 (2024): 353–363. 38526955 10.2459/JCM.0000000000001614

